# Rigor Mortis in Still-Born Children

**Published:** 1904-10-22

**Authors:** 


### RIGOR MORTIS IN STILL-BORN CHILDREN.

The difficulty of proving live birth in many cases where a charge of infanticide is preferred against the mother is very great. In fact, it is often impossible to supply evidence which the law will regard as a proof of live birth, and thus, however convinced the medical mind may be in a given instance that crime has been committed, the prisoner is usually relieved of the more serious charge. A more or less vague impression has long prevailed that rigor mortis does not occur in still-born children, and that, therefore, the presence of this condition affords definite proof of live birth. Mr. C. H. W. Parkinson,[1] on the basis of a 30 years' experience as police surgeon and in midwifery practice, has come to quite an opposite conclusion. He relates a case in which after a protracted labour the child was born dead, and with a partial development of rigor mortis, this latter condition subsequently becoming well marked. As the rigidity increased, the limbs became drawn up and flexed by it so that the body gradually assumed the form which it has *in utero*. Mr. Parkinson has observed the same thing in the bodies of other infants who were killed by craniotomy or died during birth. Limp and flaccid when delivered, there was a gradual increasing rigidity, which affected the body as already described. On the other hand, rigidity following post-natal death causes, as in adults, the limbs to stiffen in the position which they occupied at the onset of the rigidity. According to this description, therefore, rigor mortis follows ante-natal just as it follows post-natal death. It may have passed off before birth, or, occurring during labour, may obstruct delivery, or, once again, when death has only shortly preceded birth, the rigidity may only manifest itself at a subsequent stage. But, in any event, according to Mr. Parkinson, it will so change the form of the fœtus as to make it assume the shape of a fœtus *in utero*, and this appearance may thus be most valuable evidence of live birth. In one of the cases in which this was observed the body was that of a fœtus "between seven and eight months"; the others were presumably at or near full development. But the exception just mentioned is of some importance in view of the authoritative statement that "rigidity does not occur in the body of a fœtus under seven months."[2] Dr. Nutting S. Fraser[3] also records a case of rigor mortis in a still-born infant. Fœtal movements had been feebly appreciated about five hours before delivery, but on attempting to perform artificial respiration the arms were found to be rigid, and this state of the body continued until the following day.

1 Brit. Med. Jour., Sept. 24, 1904. 2 Dixon Mann: Forensic Medicine and Toxicology (Third Edit.) 3 Brit. Med. Jour., April 30, 1904.

